# Development and validation of the reproductive health literacy questionnaire for Chinese unmarried youth

**DOI:** 10.1186/s12978-021-01278-6

**Published:** 2021-11-13

**Authors:** Xuemei Ma, Yufan Yang, Qian Wei, Hong Jiang, Huijing Shi

**Affiliations:** Department of Maternal, Child and Adolescent Health, School of Public Health, Fudan University, 138 Yixueyuan Road, P.O. Box 244, Shanghai, 200032 China

**Keywords:** Reproductive health literacy, Unmarried youth, Questionnaire development, Validation

## Abstract

**Objectives:**

This study aimed to develop and validate the reproductive health literacy questionnaire for Chinese unmarried youth aged 15–24.

**Methods:**

We conducted a validity and reliability study of the questionnaire through a cross-sectional survey and test–retest analysis in four districts in Shanghai between April and June 2017. A total of 1587 participants completed a self-administered questionnaire anonymously on-site and the trained investigators conducted quality check afterwards. Sixty participants among them completed the test–retest assessment with 2 weeks interval. The reliability was determined by internal consistency, spilt-half reliability and test–retest reliability. The construct validity was assessed by confirmatory factor analysis.

**Results:**

The 58-question reproductive health literacy questionnaire for Chinese unmarried youth demonstrated high internal consistency (Cronbach’s α = 0.919), spilt-half reliability (Guttman splitting coefficient = 0.846) and test–retest reliability (correlation coefficient = 0.720). The confirmatory factor analysis showed that the construct of the questionnaire fitted well with the hypothetical model. The reproductive health literacy scores in unmarried girls aged 15–24 were higher than boys (*P* < 0.05) and college students who lived in rural areas when they were middle and high school obtained lower score than those living in cities and suburbs (*P* < 0.01).

**Conclusion:**

The reproductive health literacy questionnaire for Chinese unmarried youth demonstrated good reliability and validity, which could potentially be used as an effective evaluation instrument to assess reproductive health literacy among Chinese young people.

## Plain Language Summary

Despite growing research into evaluation structures for adolescent health literacy in the general population, there is currently a research scarcity in health literacy assessment tools for youth sexual and reproductive health. This is the first time the WHO health literacy framework has been used to construct and evaluate reproductive health literacy among Chinese youth. In this study, we described the questionnaire’s development process, reliability and validity, and utilisation among 1587 unmarried youth in Shanghai, China. Our findings indicated that the 58-item questionnaire we developed is effective to assess the reproductive health literacy in Chinese unmarried youth aged 15–24, which includes four aspects of literacy ability (accessing, understanding, appraising, and applying) to three health domains (reproductive health determinants, behavioural risk factors, and health service utilisation). The development of this tool can not only measure the reproductive health literacy level, but also be used for long-term monitoring, as well as facilitating effect evaluation of intervention. This questionnaire may also help to develop target interventions to improve reproductive health literacy of Chinese young people.

## Background

Youth aged 15–24 years are in a period of rapid matureness in reproductive function and social adaptation. With sexual consciousness awakening, secretion of sex hormones increasing and sexual impulses emerging, they need adequate reproductive health literacy to get through this key period [1]. With rapid development of the global society, youth in all societies are reaching puberty earlier but marrying later, which expands the gap between puberty and marriage [2]. Thus, unmarried youth have urgent need for accessible, quality reproductive health care. However, they are more likely to lack the knowledge, skills and ability to maintain reproductive health in low and middle income countries as they don’t have necessary information on sexual behaviour and contraception as well as inadequate understanding of negative effects of abortion, sexually transmitted diseases (STDs) and acquired immune deficiency syndrome (AIDS) [3, 4]. Notably, the long-term insufficient sexual education in China and the large population size make the reproductive health of Chinese unmarried youth an essential research focus [5].

Health literacy was first introduced in a discussion of health education at an international conference in 1974 [6]. Sørensen et al. proposed an integrated definition of health literacy in 2012, which is the competencies related to the process of accessing, understanding, appraising and applying health-related information as an asset for improving people's empowerment within the domains of healthcare, disease prevention and health promotion [7]. Improving population health literacy is regarded as a cost-effective health promotion intervention as well as an important step toward reducing health inequities [8]. With the research on health literacy continuously more specific, there is a trend that more studies emerged with aiming at specific population, such as maternal and infant health literacy [9], child health literacy [10], and patients’ health literacy [11]. However, the consensus of youth health literacy related to reproductive health measurement is still unreached. More importantly, sexual and reproductive health education in China started and developed later compared with western countries, and still in exploratory stage. Although there are limited research having explored some evaluation structures for adolescent health literacy, most of them are designed for general population and not based on a unified conceptual framework [12, 13]. Additionally, there is still a research gap about health literacy evaluation tool specifically for youth sexual and reproductive health.

In the past 27 years since the 1994 International Conference on Population and Development, where to strengthen the promotion of adolescent sexual and reproductive health was proposed, many aspects of adolescent reproductive health have substantially improved, including increasing use of contraceptives, decline of child marriage and female genital mutilation. However, there are still some challenges ahead of us, such as more STDs (including human immunodeficiency virus, HIV) and negative effect of more obesity on reproductive health, which warrants much closer attention looking forward [14]. Furthermore, as part of reproductive health literacy, early detection of sexually transmitted diseases through screening is critical for disease prevention, such as the human papillomavirus (HPV) screening test which is associated with cervical cancer [15]. Given the health literacy can be cultivated and promoted by education, it is necessary to develop a reproductive health literacy measuring tool for unmarried youth. Therefore, we proposed the concept of “reproductive health literacy” and developed it as an evaluation tool for reproductive health literacy of unmarried youth aged 15–24, to measure the reproductive health literacy among youth and to provide ideas for implementing targeted interventions to promote reproduction health.

## Methods

### Instrument development

#### Stage 1: conceptual framework and indicators generation

The conceptual framework developed by Sørensen et al. in 2012 [7] and World Health Organisation (WHO) in 2013 [16], was used in the present study, which consists of three health domains (health care, disease prevention, and health promotion) and four factors of ability to health information in each domain, including accessing, understanding, appraising, and applying. Based on this conceptual framework, a pool of 46 indicators including determinants of reproductive health in the physical and social environment (health promotion), risk factors for reproductive health (disease prevention), health services related to reproductive health problems or diseases (health care) was set up. Then, a two-round Delphi expert consultation process was carried out among 20 multi-disciplinary specialists, including researchers, doctors and teaching staff, and who working in adolescent and school health, maternal and child health, sexual and reproductive health, and health education. They were required to evaluate the content representativeness, health literacy relevance, and evaluation feasibility of each indicator [17]. As a result, 45 reproductive health literacy indicators were identified by consensus, as shown in Table 1.

**Table 1:** Indicators of reproductive health literacy and number of questionnaire items in each ability factor for unmarried youth in three health domains

	Determinants of reproductive health (Health promotion)		Risk factors for reproductive health (Disease prevention)		Health service utilisation (Health care)	
	Indicators	No. of items	Indicators	No. of items	Indicators	No. of items
Accessing	1. Access to knowledge of AIDS and relevant national policy and regulation 2. Access to information on promoting reproductive health 3. Access to information about gender identity and sexual orientation	3	1. Acquire information about unprotected sexual behaviour 2. Acquire knowledge about adolescent health care (e.g. weight control, sleep, menstrual care) 3. Acquire information on the health hazards of sexual assault and how to prevent sexual assault	3	1. Acquire medical information on safe abortion via good sources 2. Acquire information on medical institutions about AIDS and HIV testing 3. Acquire information about how to get free contraceptives 4. Acquire knowledge about clinical symptoms of sexually transmitted diseases, methods for prevention and medical institutions 5. Acquire information about the prevention and treatment institutions for adolescent sexual health problems	7
Understanding	1. Understand sexual development events and their meaning 2. Understand how to distinguish the differences between friendship, love and infatuation when getting along with others, and understand the responsibility and ethics of marriage and family	4	1. Understand the risk factors for sextual behaviour of being affected AIDS (unprotected sextual activity with multiple partners) 2. Understand the health hazards of unprotected sextual activity before marriage 3. Understand that the unhealthy lifestyle is a risk factor for amenorrhea and menstrual disorders 4. Understand the concept of unprotected sex 5. Understand the high-risk situations or scenarios of sexual assault and that the perpetrator may be an acquaintance	6	1. Understand the basic concepts and transmission routes of AIDS and be aware of the asymptomatic incubation period 2. Understand the basic concepts and symptoms of women's menstrual problems (abnormal menstruation, dysmenorrhea) 3. Understand the dual-protective effects of condoms (contraception and avoiding sexually transmitted diseases) 4. Understand the hazards of abortion and repetitive miscarriage 5. Understand the basic manifestations of abnormal male external genitalia (foreskin and phimosis) 6. Understand the main symptoms of sexually transmitted infections 7. Understand the scenarios in which it is possible to become pregnant 8. Understand the meaning of HIV test results and the window period of AIDS 9. Understand the basic concepts of puberty stunting	13
Appraising	1. Analyse and evaluate risk factors of adolescent reproductive health from the media consciously 2. Be able to identify all kinds of unhealthy information and establish a positive, healthy and responsible sexual mores	2	Be able to analyse possible scenarios of unprotected sexual behaviour of young people	1	1. Be able to care about puberty development, communicate in time, and take the initiative in seeking help when in doubt 2. Be able to distinguish the information on managing unintended pregnancy in media 3. Do NOT discriminate against AIDS patients or HIV-infected 4. Be able to judge the reliability of AIDS information consciously	4
Applying	Be able to communicate with professional counselors and doctors rather than blindly defining yourself, when confused about your sexual orientation or gender identity	1	1. Understand how to protect yourself and partner during sex 2. Dare to refuse and take appropriate measures to protect yourself when you are sexually assaulted or harassed 3. Be able to retain evidence (not bathing immediately) and seek social and medical help in time after being sexually assaulted 4. Be able to avoid exposures to high-risk situations of being sexual assaulted 5. Manage to avoid unprotected sex before marriage and make healthy decisions	7	1. Be able to detect menstrual abnormalities in time and be willing to seek medical help 2. Be able to detect genital inflammation in time and seek medical help 3. Go to a regular hospital for examination and treatment if suspect pregnant 4. Manage to detect the symptoms of sexually transmitted infections in time and seek medical help 5. Voluntarily counsel and test for AIDS if suspect infection 6. Be able to detect delayed puberty in time and be willing to seek medical help	7

#### Stage 2: questionnaire development

The final questionnaire was developed based on the 45 indicators. Firstly, in general, each indicator was directly transformed into one item and some indicators were converted into two items through discussion within the research group. Secondly, feedbacks from 4 experts with extensive professional experience were collected via email to modify the questionnaire. Thirdly, a total of 30 volunteers, including 10 public health professionals and 20 unmarried youth, were invited to complete the questionnaire and provide feedback. Then, based on their feedback, we adjusted the statements and improved the questionnaire language to avoid ambiguity and make the questionnaire more reader-friendly. Lastly, a questionnaire with 58 items on reproductive health literacy for unmarried youth was formed, which covered six topics: physiological and psychological development during adolescence, personal health care during adolescence, heterosexual relationship and sexual behaviour, prevention and response to sexual harassment and sexual abuse, prevention of AIDS and STDs, and prevention and response to unintended pregnancy. The questionnaire was also involved with four aspects of literacy ability (accessing, understanding, appraising, and applying) to reproductive health determinants, behavioural risk factors, and health service utilisation, as shown in Table 1.

The scoring system was as follows: (1) 4 points for the correct answer to the two-category multiple-choice items and 0 point for the wrong answer; (2) In the graded multiple-choice items, 0, 1, 2, 3, and 4 points were assigned to the answers of “I don’t know”, “very difficult”, “difficult”, “easy” and “very easy”, respectively; (3) When multiple-choice items were scored, each option was treated as a true/false item. If there is "n" options, the correct score for each option was 4/n and the total score of this item was the sum of the scores for each option. If the answer is “don’t know”, 0 point was assigned directly. According to this rule, the scores of each item were uniformly processed into continuous variables in the range of 0 ~ 4.

### Validation of the questionnaire

#### Study participants and data collection

A cross-sectional survey was conducted with multi-stage sampling method in four districts in Shanghai from April to June 2017, including Huangpu District, Jiading District, Songjiang District and Pudong District of Shanghai. The inclusion criteria were as follows: (1) students of grade one and two in general public high schools; (2) undergraduate students in the first three years from general universities and junior colleges; (3) unmarried youth who have basic Chinese reading skills and been informed consent. The multi-stage sampling method was carried out as follows. Two general public schools in each district and two institutions of higher education (one general university and one junior college) both in Songjiang District and Pudong District were included. Then, using a stratified random sampling method, two classes from each grade of each school/college were chosen, and 30 students (15 girls and 15 boys) from each class were sampled by a systematic sampling method. Finally, a total of 1606 participants were recruited to complete a self-administered questionnaire anonymously on-site and the trained investigators conducted quality check afterwards. In order to evaluate test–retest reliability, 60 students from a high school in Songjiang District were invited to complete the questionnaire again two weeks later. Data on demographics and reproductive health status were also collected from the participants, including age, gender, place of household registration, parents’ education levels, family income, etc. This study was approved by the Ethics Committee of School of Public Health, Fudan University (IRB#2016-11-0604).

#### Item analysis

The measuring indicators used for item analysis included degree of difficulty, degree of discrimination, and internal consistency analysis. Items that were too simple, undistinguished and had no contribution to internal consistency were deleted. Accordingly, if the item met the following three conditions at the same time: degree of difficulty > 0.8; discrimination < 0.2; internal consistency became better after deleting the item; then the item was deleted.

After item analysis, the degree of difficulty for items ranged 0.27–0.89, and the degree of discrimination ranged 0.155–0.572, and the correlation coefficients were statistically significant. Among them, 25.86% of items had the degree of difficulty > 0.7, that was, about one quarter of the items were relatively not difficult. As a result, no items were deleted.

#### Reliability and validity test

All the items in the reproductive health literacy questionnaire were divided into three parts for reliability and validity evaluation according to their first-level indicators: Health Care, Disease Prevention and Health Promotion [18].

Reliability was measured by the internal consistency, split-half reliability and test–retest reliability [19]. The internal consistency was measured with Cronbach’s α coefficient, and the split-half reliability was measured with the Guttman half-coefficient between odd and even items. Additionally, test–retest reliability was measured with the correlation coefficient between the twice results completed by the 60 high school students with a two-week interval. Each of the values greater than 0.70 indicated acceptable reliability [19, 20].

Validity was measured by the content validity and structural validity. The content validity was ensured by the repeated revisions from the international advanced health literacy conceptual model, the consultation of the experts, and practical experience of the questionnaire. The confirmatory factor analysis (CFA) was used to verify the construct validity [21]. The analysis was conducted separately for each domain of the questionnaire (health control, disease prevention, health promotion) to match the four domains of “acquisition”, “understanding”, “evaluation” and “application” in the conceptual framework. The model fit was evaluated by the key indicators of root mean square error of approximation (RMSEA), goodness-of-fit index (GFI) and adjusted goodness-of-fit index (AGFI), with which < 0.08, > 0.9, > 0.9, respectively, indicated “relatively good” fit [18, 22].

### Statistical analysis

The returned questionnaires were entered into database by using Epidata 3.1, and the invalid questionnaires (the missing data in a questionnaire more than 20%) were deleted. The scores of each item in the reproductive health literacy questionnaire were calculated in Excel 2016 while reliability analyses and other parametric tests were computed in SPSS 21.0. Descriptive statistics of the participants’ characteristics, correlation analysis, t-test and one-way analysis of variance (ANOVA) were conducted to describe the sociodemographic characteristics and reproductive health literacy scores, to understand the relationship among various domains of health literacy, and to estimate the potential risk factors related to health literacy. Additionally, CFA was conducted with maximum likelihood estimation by using AMOS 21.0. The significance level was set at *P* < 0.05.

When scoring each item for reproductive health literacy, the indicator score was the average score of corresponding items if one indicator corresponded to multiple items. Then the total score was the indicator score multiplied by its corresponding weight which was assessed during Delphi consultation. The final standardised total score (out of 100) was calculated as follows: final total score = ∑ (each indicator score × corresponding weight) × 100/4. A higher score indicated higher reproductive health literacy.

## Results

Results of the validation study of the 58-item reproductive health literacy questionnaire for Chinese unmarried youth using a cross-sectional survey are presented below.

### Sociodemographic characteristics of participants and descriptive results for the questionnaire

A total of 1587 valid questionnaires were collected from schools in four districts in Shanghai. As shown in Table 2, gender distribution of the participants was balanced, and the age ranged from 16 to 25 years old, with 94.9% in the 16–21 age group. Nearly 90% of the high school students’ household registration was in Shanghai, with the non-Shanghai households of college students accounted for more than 50%. Similarly, more than 70% of high school students and only about 40% of college students lived in large cities during junior and high school age. In addition, the monthly income per capita of most core family was 4500 ~ 7500 RMB, accounting for 35.0%, and 28.1% of the families got 7500 ~ 12,500 RMB monthly income per capita.

**Table 2:** Demographic and socio-economic characteristics of participants and average scores of reproductive health literacy

	Total n (%)	High school student n (%)	Score Mean ± SD	F/t (P)	College student n (%)	Score Mean ± SD	F/t (P)
Gender	1587	960		10.661 (0.001)^a^	627		6.122 (0.013)^a^
Male	791 (49.8)	479 (49.9)	59.87 ± 16.21		312 (49.8)	58.30 ± 18.83	
Female	796 (50.2)	481 (50.1)	63.15 ± 13.72		315 (50.2)	62.46 ± 14.80	
Age	1564	942		0.022 (0.883)	622		1.982 (1.139)
16 ~ 18	945 (60.4)	932 (98.9)	61.53 ± 15.14		13 (2.1)	62.10 ± 10.61	
19 ~ 21	540 (34.5)	10 (1.1)	62.23 ± 8.04		530 (85.2)	60.84 ± 17.20	
22 and above	79 (5.1)	–	–		79 (12.7)	56.84 ± 16.07	
Place of household registration	1576	958		0.308 (0.579)	618		1.637 (0.201)
Shanghai	1124 (71.3)	859 (89.7)	61.48 ± 15.17		265 (42.9)	59.44 ± 15.92	
Other provinces	452 (28.7)	99 (10.3)	62.36 ± 13.99		353 (57.1)	61.22 ± 17.97	
Living area before age of 7	1575	952		1.825 (0.141)	623		1.475 (0.220)
Big cities	916 (58.2)	667 (70.1)	62.31 ± 17.94		249 (40.0)	59.19 ± 17.89	
Medium and small cities	338 (21.5)	119 (12.5)	60.17 ± 16.17		219 (35.2)	62.04 ± 17.06	
Towns and outskirts	255 (16.2)	156 (16.4)	59.55 ± 14.49		99 (15.9)	61.34 ± 14.61	
Countryside	66 (4.2)	10 (1.1)	61.19 ± 15.88		56 (9.0)	58.28 ± 17.16	
Living area during junior and high school age	1572	951		2.324 (0.074)	621		4.268 (0.005)
Big cities	962 (60.6)	701 (73.7)	62.32 ± 14.97		261 (42.0)	60.70 ± 17.03	
Medium and small cities	370 (23.3)	122 (12.8)	59.39 ± 15.48		248 (39.9)	61.25 ± 16.93	
Towns and outskirts	212 (13.4)	123 (12.9)	59.43 ± 15.13		89 (14.3)	60.76 ± 15.60	
Countryside	28 (1.8)	5 (0.5)	64.31 ± 13.25		23 (3.7)	48.13 ± 20.77	
Family monthly income per capita (in RMB)	1556	951		0.443 (0.777)	605		5.515 (0.272) ^a^
< 1500	40 (2.6)	10 (1.1)	57.77 ± 15.21		30 (5.0)	59.97 ± 13.29	
1500 ~ 4500	299 (19.2)	184 (19.3)	61.06 ± 15.08		115 (19.0)	62.83 ± 16.20	
4500 ~ 7500	544 (35.0)	335 (35.2)	61.40 ± 14.80		209 (34.5)	60.51 ± 15.19	
7500 ~ 12,500	438 (28.1)	281 (29.5)	62.17 ± 14.66		157 (26.0)	59.27 ± 17.71	
> 12,500	235 (15.1)	141 (14.8)	62.43 ± 15.58		94 (15.5)	60.42 ± 19.84	
Family socio-economic status*	1582	958		2.455 (0.117)	624		0.412 (0.512)
High (Green score > Median)	786 (49.7)	473 (49.4)	62.34 ± 14.57		313 (50.2)	60.90 ± 17.20	
Low (Green score ≤ Median)	796 (50.3)	485 (50.6)	60.81 ± 15.48		311(49.8)	60.02 ± 16.92	

^a^Non-parametric tests (Mann–Whitney U test or Kruskal–Wallis test). *Family socio-economic status was determined by Green score [23], which was calculated based on maternal and paternal occupation and education. Green score = (paternal education × 0.7 + paternal occupation × 0.4 + maternal education × 0.7 + maternal occupation × 0.4)/2

After comparing the average reproductive health literacy score in different socio-demographic groups, score differences in sex were significant both in high school students and college students. Among 960 high school students, girls average scored 3.28 higher than boys (U = 10.661, *P* = 0.001), and among 627 college students, girls average scored 4.16 higher than boys (U = 6.122, *P* = 0.013). Additionally, the living area during junior and high school age was associated with the scores of reproductive health literacy of college students (*F* = 5.023, *P* < 0.01). Further analysis between either two groups showed the score of the students who lived in rural areas during junior and high school age was lower than that of students who lived in the large cities (*P* = 0.001), small and medium-sized cities (*P* < 0.001), and rural suburbs (*P* = 0.002).

Overall, the P_50_ (P_25_, P_75_) of the total score of the reproductive health literacy among 1587 participants was 64.67 (53.30, 72.19). As shown in Table 3, of three domains, total score in disease prevention was highest, with median of 73.77, whereas total in health promotion was lowest, with median of 57.42. On the other hand, compared with other 3 ability aspects, the ability of applying was highest. Additionally, the results showed significant differences across scores in three domains (χ^2^ = 409.8, *P* < 0.01). After compared in pairs, these scores indicated significant differences between either two of them, with the highest score in disease prevention and the lowest score in health promotion.

**Table 3:** Total and different ability indicators of all three domains in reproductive health literacy among 1587 participants, P_50_ (P_25_, P_75_)

	Health care	Disease prevention	Health promotion
Accessing	55.48 (40.31, 69.30)	66.77 (49.38, 75.00)	56.26 (42.19, 70.29)
Understanding	55.14 (42.00, 68.21)	74.74 (58.52, 86.70)	50.00 (30.08, 69.92)
Appraising	53.73 (41.26, 62.66)	75.00 (50.00, 100.00)	62.12 (49.24, 74.24)
Applying	79.67 (64.20, 89.53)	81.27 (60.21, 92.06)	100.00 (0.00, 100.00)
Total score	60.55 (49.00, 69.67)	73.77 (59.89, 82.14)	57.42 (45.41, 67.40)

### Reliability

As shown in Table 4, generally, the Cronbach’ α coefficient of total questionnaire and the three domains (health control, disease prevention, health promotion) ranged 0.493–0.919, and the Guttman splitting coefficient ranged 0.469–0.846, indicating the good internal consistency of the questionnaire. However, the values for the health promotion were relatively lower, which was due to fewer items in this domain. The correlation coefficients of the questionnaire completed by 60 participants’ twice in two weeks ranged 0.495–0.772, which indicated good test–retest reliability.

**Table 4:** Reliability of total and three domains of the reproductive health literacy questionnaire for Chinese unmarried youth

Reliability	Total (n = 58)	Health care (n = 31)	Disease prevention (n = 17)	Health promotion (n = 10)
Cronbach’s α coefficient	0.919	0.868	0.853	0.493
Guttman splitting coefficient	0.846	0.772	0.833	0.469
Test–retest coefficient	0.720^**^	0.772^**^	0.621^**^	0.495^**^

n is the number of items in each domain. ^**^P < 0.01

### Validity

Content validity was ensured by the scientific developing process of the reproductive health literacy questionnaire. Firstly, an extensive literature review was conducted to identify the main reproductive health problems in the 15 ~ 24 age group, followed by an on-site discussion within 20 experts to develop 45 indicators based on WHO’s health literacy framework. Secondly, 20 professionals were invited to give feedback repeatedly to improve the indicators and revised into 46. Thirdly, an original version of the questionnaire was developed based on the indicators, and experts and volunteers were invited to improve it through professional suggestions and language changes.

### Construct validity

The results of the confirmatory factor analysis (CFA) showed a relatively good fit of all the four ability factors within three domains of reproductive health literacy (Table 5).

**Table 5:** Model fit indices of the reproductive health literacy questionnaire for Chinese unmarried youth by CFA

Indices of model fit	Health care^*^ (n = 31)	Disease prevention (n = 17)	Health promotion (n = 10)
χ^2^/*df*	5.988	6.539	8.259
RMSEA	0.056	0.059	0.068
GFI	0.908	0.942	0.966
AGFI	0.885	0.922	0.938
CFI	0.865	0.926	0.820

n is the number of questionnaire items in each domain. ^*^Modification Indices. *CFA* confirmatory factor analysis, *RMSEA* root mean square error of approximation, *GFI* goodness-of-fit index, *AGFI* adjusted goodness-of-fit index, *CFI* comparative fit index

## Discussion

The 58-question reproductive health literacy questionnaire for Chinese unmarried youth was developed for evaluating reproductive health literacy among Chinese young people. The validation study was carried out among 1587 students from 12 high schools and colleges in four districts of Shanghai, which was a representative sample of Shanghai. The range of the reproductive health score is between 0 and 100, with median of 64.67, and a higher score indicates higher reproductive health literacy level. Psychometric analysis results indicated that it has good reliability and validity and could be a useful instrument for assessing reproductive health literacy for unmarried youth in the Chinese context.

The development of reproductive health literacy questionnaire for Chinese unmarried youth is in line with the opinion from Nutbeam who proposed that the measurement of health literacy would be best achieved where content and context were well defined [24]. This study was based on the conceptual framework of health literacy [7], that is to say, reproductive health literacy integrated the content of medical services and public health, formed by three domains (medical services, disease prevention and health promotion) and four abilities (acquisition, understanding, evaluation and application). The application of this conceptual framework provides a theoretical basis for the development of instruments for assessing reproductive health literacy, with which we developed and applied the first measurement in China for assessing reproductive health literacy among young people.

The psychometric evaluation of the reproductive health literacy questionnaire for Chinese unmarried youth produced plausible results. The overall 58-question questionnaire was reliable, demonstrated by high internal consistency, spilt-half reliability and test–retest reliability (all coefficients > 0.7). For the three domains, all reliability coefficients were over 0.6 which was considered as acceptable reliability [25], except for the health promotion. It may be due to the less amount of questions in this domain, therefore, we recommend that the questionnaire should be administered as a whole when applying in the future studies. The results of confirmatory factor analysis suggested that the construct of the questionnaire fitted well with the theoretical model, represented an acceptable fit [22, 26]. On the other hand, we applied various methods to ensure the content validity of the questionnaire, including literature review, professional consultation and exiting evidence reference, as well as application of the health literacy integration conceptual framework [7]. In the meantime, both experts and unmarried youth were asked to provide feedback to help us improve the questionnaire’s content validity as well as language adjustments to make the questionnaire more reader-friendly.

The total reproductive health literacy score among youth in our study was around 60, indicating that the overall reproductive health literacy of the unmarried youth in Shanghai is at an intermediate level. Our study found that the unmarried youth obtained the highest score in “disease prevention”, and the lowest in “health promotion”, which suggests that unmarried youth in China have a poor understanding of the social determinants of reproductive health. The reasons may be that they do not recognise the importance of reproductive health or they cannot effectively identify the social determinants of reproductive health in daily life, which reminds that the social environment and policy system for the reproductive health of unmarried youth need to be further optimised and improved [27]. Additionally, we found that youth scored lower in the competences of accessing, understanding and appraising, compared with the competence of applying, indicating that a comprehensive health literacy intervention is needed to empower youth to access, understand and appraise health information [4]. Rather than the “knowledge, attitude and practice” model, health literacy covers the acquisition and evaluation of health information. Also, we found that youth scored lower in these two ability factors, indicating young people still lack access to health information and authorities should establish more professional platforms to reduce barriers in acquiring reproductive health information and provide professional and accessible information to the public via audience-friendly ways [28].

The main social determinants that affect the scores of high school and college students' reproductive health literacy were gender and the living area in junior and high school. We found that unmarried girls aged 15–24 had higher scores than boys and the difference is statistically significant, which is consistent with other studies on health literacy among Chinese young people [29–32]. It may be due to the fact that girls are sexually mature earlier than boys and are more concerned about their own health. Another explanation could be that society often places higher moral expectations on females due to the differences in gender norms, causing girls to receive more information about reproductive health at a younger age than boys [33]. Additionally, the living area during junior and high school was associated with the scores of reproductive health literacy among college students. Specifically, college students who lived in rural areas when they were at middle and high school age scored lower than those living in large cities, small and medium-sized cities and suburbs. This may be because of the fact that middle and high school stage is a critical phase for sexual development, with relatively high plasticity and health awareness [34]. Furthermore, rural areas have a more conservative culture, a lower level of economic development and a relative lack of health resources as compared to cities, all of which may contribute to the difference in scores between rural and urban youth. However, we did not find significant effects of family social status (parents’ occupation and education) and family income per capita on the reproductive health score among unmarried youth, suggesting that reproductive health literacy level in the 15–24 age group is hardly influenced by the family, which is inconsistent with the results from Xu et al. [35]. It may be that reproductive health is different from other health areas, and it is still a private and sensitive topic in the Chinese family environment [5]. The acquisition and exchange of reproductive health information may come more from school and peer education than family [36, 37]. Thus, future reproductive health promotion interventions should be considered for implementation in the school setting.

The development and validation of an appropriate measurement is fundamental and essential for youth reproductive health literacy research. To the best of our knowledge, this is the first study proposing the concept of reproductive health literacy and also the first time developing and evaluating reproductive health literacy questionnaire for unmarried youth in China. Additionally, this study suggests that improving reproductive health literacy can be a good way to promote reproductive health. Improving the reproductive health literacy of unmarried youth will help reduce and prevent reproductive health problems in this age group, improve the utilisation rate of reproductive health services and compliance of reproductive health related diseases treatment, and thereby promote the reproductive health and decrease social burden of related disease [4].

However, some limitations of this study should be concerned. First, the reproductive health literacy presented in this study only included unmarried youth in schools and colleges but not the same age population outside schools. Second, since the participants were all from Shanghai, which are not representative enough for the whole country, further studies are needed to generalise this measurement in other regions and settings of China. Third, response bias cannot be avoided as the questionnaire is based on self-reporting.

## Conclusion

This is the first time developing the concept and evaluating domains of reproductive health literacy based on the WHO health literacy framework. The reproductive health literacy questionnaire has demonstrated good reliability and validity among Chinese youth aged 15–24. The development of this tool can not only measure the reproductive health literacy level, but also be used for long-term monitoring, as well as facilitating effect evaluation of intervention. This questionnaire may also help to develop target interventions to improve reproductive health literacy of Chinese young people.

## Data Availability

The datasets analysed during the current study are available from the corresponding author on reasonable request.

## Abbreviations

STDs: Sexually transmitted diseases
AIDS: Acquired immune deficiency syndrome
HIV: Human immunodeficiency virus
HPV: Human papillomavirus
WHO: World Health Organisation
CFA: Confirmatory factor analysis
RMSEA: Root mean square error of approximation
GFI: Goodness-of-fit index
AGFI: Adjusted goodness-of-fit index
CFI: Comparative fit index
ANOVA: Analysis of variance
RMB: Renminbi (Chinese Yuan)
